# Research and Capacity Building for Control of Neglected Tropical Diseases: The Need for a Different Approach

**DOI:** 10.1371/journal.pntd.0001020

**Published:** 2011-05-31

**Authors:** Thomas Kariuki, Richard Phillips, Sammy Njenga, Ole F. Olesen, Paul R. Klatser, Riccardo Porro, Sarah Lock, Maria Hermínia Cabral, Martina Gliber, Detlef Hanne

**Affiliations:** 1 Institute of Primate Research, National Museums of Kenya, Nairobi, Kenya; 2 KNUST School of Medical Sciences, Komfo Anokye Teaching Hospital, Kumasi, Ghana; 3 Kenya Medical Research Institute, Centre for Microbiology Research, Nairobi, Kenya; 4 Neglected Infectious Diseases, Infectious Diseases Unit, DG Research, European Commission, Brussels, Belgium; 5 Royal Tropical Institute (KIT), KIT Biomedical Research, Amsterdam, The Netherlands; 6 Fondazione Cariplo, Milano, Italy; 7 The Nuffield Foundation, London, United Kingdom; 8 Fundação Calouste Gulbenkian, Lisbon, Portugal; 9 Fondation Mérieux, Lyon, France; 10 Volkswagen Foundation, Hanover, Germany; The George Washington University, United States of America

The neglected tropical diseases (NTDs) are a group of chronic disabling infections affecting more than 1 billion people worldwide, mainly in Africa and mostly those living in remote rural areas, urban slums, or conflict zones. By considering the NTDs together, it is clear that they threaten the health of the poorest to a similar extent as HIV/AIDS, malaria, and tuberculosis (TB) [1]. Beyond their negative direct impact on health, NTDs also fuel the vicious circle of poverty and stigma that leaves people unable to work, go to school, or participate in family and community life. Whilst “the big three” infections have caught the world's attention, these other disabling and sometimes fatal infectious diseases in Africa have until very recently been receiving relatively little attention from donors, policymakers, and public health officials. Yet NTD control represents a largely untapped development opportunity to alleviate misery and poverty in the world's poorest populations, and therefore has a direct impact on the achievement of the Millennium Development Goals.

The impact of NTDs on health and economy is now increasingly discussed in international fora, e.g., recently in a series of articles in *The Lancet* at the beginning of 2010 ([2], http://www.thelancet.com/series/neglected-tropical-diseases). However, the discussion is clearly dominated by scientists from the industrialized (“Northern”) countries. While the African continent is particularly hard hit by NTDs, the African scientific community has so far been poorly represented during global priority setting for research on NTDs. Also, in most cases, the importance of scientific capacity building in endemic developing countries that would guarantee ownership, support, and sustainability of control programs is neglected. In this article, we would therefore like to summarize the deliberations and recommendations of two workshops held in Bamako and Lisbon in 2008 and 2010, respectively, where about 50 researchers from sub-Saharan Africa discussed their views on research and capacity requirements for the control of NTDs. The workshops were organized under the framework of the European Foundation Initiative for African Research into Neglected Tropical Diseases (EFINTD, http://www.ntd-africa.net/), which consists of five European foundations, and aims to combat NTDs by offering funding for postdoctoral fellows from sub-Saharan Africa to pursue scientific careers in their home continent. The initiative also facilitates the creation of collaborative scientific networks linking researchers within Africa, and between Northern and African scientists.

It was interesting to observe that the workshop participants identified the same scientific challenges that must be overcome for control of NTDs as their colleagues from the North: the need for the development of new diagnostic tools, vaccines, and drugs; the development of efficient drug delivery systems; detailed epidemiological investigations; and community-based implementation research. All these might reflect the intensive networking between the scientists and general agreement on the urgent imperative to investigate on these topics, but it might also be the result of the general domination of Northern scientists in these discussions. At the same time, there were some distinct differences expressed by the African scholars: the need to focus more strongly on the short-term applicability of research and its relevance to national and regional health problems in Africa, as well as its benefits to the local population. Instead of concentrating on pure basic research and scientific impact factors, more research efforts should be dedicated to operational research and better application of existing tools in the health system. In addition, the African scholars highlighted the lack of sufficient funds from individual funding organizations available for African institutions (for infrastructure support as well as project funding) to enable them to work efficiently in their home countries. Indeed, this was noted to be a major factor hindering African scholars trained abroad from returning to their home countries to pursue careers in health research.

In addition to the paucity of financial resources for conducting research, the African scientists were unanimous in supporting the institution of career development schemes (e.g., mentorship programs, project management courses, proposal-writing workshops, language training, and networking opportunities, such as workshops and conferences). The African researchers reiterated that the burden of supporting research for NTDs was not the sole purview of Northern donors, but that the governments of their home countries should also take some responsibility for providing adequate infrastructure and job opportunities. The EFINTD was seen as a promising and novel contribution to long-term capacity development, and as a platform for initiation of networking schemes. However, it was suggested that better, faster, and more focused outcomes could be achieved if a coordinated approach involving other funding organizations was in place; this could provide much needed support to large research infrastructures or permit extensions of programs, which would help to sustain research activities in sub-Saharan Africa. The selection process adopted by the EFINTD was seen as very positive: it was one of the few opportunities for junior researchers to apply for their own funding, and the intense selection through a final conference with presentation and interviews was seen as most appropriate, because it was one of the very few occasions where they received direct feedback on their ideas from a panel of internationally recognized experts.

The deliberations raised above pose a serious question to research funders in general: what is the role of funding organizations when trying to promote careers of young scientists from developing countries? The traditional way of funding large cooperative research projects between partners in the North and partners in the South is not the only solution, because they are usually dominated by the Northern scholars and often result in brain drain from the South. At the same time, without the support of international experts, most junior scholars from sub-Saharan Africa would not be able to develop their careers, because they need their expertise, access to state-of-art facilities, and networks. For most African research institutions, capacity building is also needed at the institutional level. Even when funding is granted directly to research institutions in the South, the scholars may not be able to exploit the full potential of the resources provided because the institutions may not have the critical scientific mass and equipment to execute the proposed programs, or there may not be sufficient administrative capacity available to deal with these sometimes very large and complex projects. This lack of institutional administrative capacity has been a deterrent for funding organizations that prefer to lodge their finances with Northern partners. A further aspect of this is that funding organizations have their own rules for monitoring and evaluation which sometimes differ immensely (not to mention the sometimes very complicated application procedures). Combined with the usual high expectations on the side of the donors, there is accordingly an increased risk of failure compared to the situation in the North.

These thoughts and ideas are leading to two conclusions for organizations engaged in funding research in sub-Saharan Africa, which are usually overlooked: 1) funding organizations should communicate more amongst each other to really complement, coordinate, and harmonize efforts, without losing sight of accepted good practices or rigorous (peer) review of both the science and the instruments of financial administration. Ideally, they should standardize and simplify application and reporting procedures, and 2) they should be involved more strongly in the projects from the beginning, not in a way of patronizing or controlling, but rather through genuine partnerships. If funding organizations are willing to become partners rather than mere providers of funds, then they need to acquire the necessary knowledge of the scientific fields and regions they are investing in. It will require more investment in human resources in disease endemic countries, a focused approach to assist African institutions in developing their own capacities, and a new mind-set and willingness to be much more than just donors and detached grant administrators.
